# Radiomic Analysis in Contrast-Enhanced Spectral Mammography for Predicting Breast Cancer Histological Outcome

**DOI:** 10.3390/diagnostics10090708

**Published:** 2020-09-17

**Authors:** Daniele La Forgia, Annarita Fanizzi, Francesco Campobasso, Roberto Bellotti, Vittorio Didonna, Vito Lorusso, Marco Moschetta, Raffaella Massafra, Pasquale Tamborra, Sabina Tangaro, Michele Telegrafo, Maria Irene Pastena, Alfredo Zito

**Affiliations:** 1 Struttura Semplice Dipartimentale di Radiologia Senologica, I.R.C.C.S. Istituto Tumori “Giovanni Paolo II”, Viale Orazio Flacco 65, 70124 Bari, Italy; d.laforgia@oncologico.bari.it; 2 Struttura Semplice Dipartimentale di Fisica Sanitaria, I.R.C.C.S. Istituto Tumori “Giovanni Paolo II”, Viale Orazio Flacco 65, 70124 Bari, Italy; annarita.fanizzi.af@gmail.com (A.F.); v.didonna@oncologico.bari.it (V.D.); p.tamborra@oncologico.bari.it (P.T.); 3 Dipartimento di Economia e Finanza, Università degli Studi di Bari “Aldo Moro”, Largo Abbazia S. Scolastica, 70124 Bari, Italy; francesco.campobasso@uniba.it; 4 Dipartimento Interateneo di Fisica, Università degli Studi di Bari “Aldo Moro”, Via Giovanni Amendola, 165/a, 70126 Bari, Italy; roberto.ballotti@uniba.it; 5 Istituto Nazionale di Fisica Nucleare, Sezione di Bari, Via Giovanni Amendola, 165/a, 70126 Bari, Italy; Sonia.Tangaro@ba.infn.it; 6 Unità Operativa Complessa di Oncologia Medica, I.R.C.C.S. Istituto Tumori “Giovanni Paolo II”, Viale Orazio Flacco 65, 70124 Bari, Italy; vitolorusso@me.com; 7 Unità Operativa Semplice Dipartimentale Radiodiagnostica ad Indirizzo Senologico, Azienda Ospedaliero-Universitaria Consorziale Policlinico, Piazza Giulio Cesare 11, 70124 Bari, Italy; marco.moschetta@uniba.it (M.M.); mikitele@hotmail.it (M.T.); 8 Dipartimento di Scienze del Suolo, della Pianta e degli Alimenti, Università degli Studi di Bari Aldo Moro, 70121 Bari, Italy; 9 Unità Operativa Complessa di Anatomia Patologica, I.R.C.C.S. Istituto Tumori “Giovanni Paolo II”, Viale Orazio Flacco 65, 70124 Bari, Italy; m.pastena@oncologico.bari.it (M.I.P.); a.zito@oncologico.bari.it (A.Z.)

**Keywords:** breast cancer, histological outcome, Her2 positive, triple negative, radiomic analysis, correlation, prediction

## Abstract

Contrast-Enhanced Spectral Mammography (CESM) is a recently introduced mammographic method with characteristics particularly suitable for breast cancer radiomic analysis. This work aims to evaluate radiomic features for predicting histological outcome and two cancer molecular subtypes, namely Human Epidermal growth factor Receptor 2 (HER2)-positive and triple-negative. From 52 patients, 68 lesions were identified and confirmed on histological examination. Radiomic analysis was performed on regions of interest (ROIs) selected from both low-energy (LE) and ReCombined (RC) CESM images. Fourteen statistical features were extracted from each ROI. Expression of estrogen receptor (ER) was significantly correlated with variation coefficient and variation range calculated on both LE and RC images; progesterone receptor (PR) with skewness index calculated on LE images; and Ki67 with variation coefficient, variation range, entropy and relative smoothness indices calculated on RC images. HER2 was significantly associated with relative smoothness calculated on LE images, and grading tumor with variation coefficient, entropy and relative smoothness calculated on RC images. Encouraging results for differentiation between ER+/ER−, PR+/PR−, HER2+/HER2−, Ki67+/Ki67−, High-Grade/Low-Grade and TN/NTN were obtained. Specifically, the highest performances were obtained for discriminating HER2+/HER2− (90.87%), ER+/ER− (83.79%) and Ki67+/Ki67− (84.80%). Our results suggest an interesting role for radiomics in CESM to predict histological outcomes and particular tumors’ molecular subtype.

## 1. Introduction

In females, breast cancer is currently the disease with the highest social impact in the world. In fact, it is the most widespread and lethal tumor among women even though the survival index has increased in the last 20 years due to early diagnosis and generally better treatments [1].

Mammography is the most-adopted diagnostic technique for screening programs as well as the first cause for mortality reduction. Nevertheless, it suffers some limitations, such as in the case of a dense breast.

For these reasons, other techniques such as Magnetic Resonance (MR) are associated with mammographic survey in selected cases [2]. With the introduction of Full-Field Digital Mammography (FFDM), other techniques such as Tomosynthesis or Contrast-Enhanced Spectral Mammography (CESM) were shown to be effective for detection and correct staging of cancer in dense breasts [3,4]. Specifically, CESM stands out as an especially complete method compared to the others as it is capable of simultaneously providing mammogram-like images, associated with contrasting images capable of providing data on the neo-angiogenesis of lesions.

Recent studies show that CESM is a useful diagnostic tool for breast cancer therapeutic planning because it provides preoperative staging with an accuracy not inferior to magnetic resonance imaging [5]. Its intrinsic characteristics are especially effective when MR examination cannot be performed. However, diagnosis by CESM can still be considered operator dependent.

In image processing, feature extraction plays an important role. In fact, the quantitative information extracted from images by means of physical and mathematical methods completes those detected by the qualitative survey carried out by the operator [6]. Image analysis, known as radiomics, is an emerging discipline in cancer studies and is often used in models aimed at characterizing tissues and recognizing lesions of various kinds in biomedical images. [7,8]. Radiomic analysis can better study the variations of heterogeneity inside the neoplasm compared to biopsy, in a non-invasive way and over a long period of time: this aspect is extremely important and is being studied in more methods of breast care because it could allow for better evaluation of clinical efficacy of oncological therapies and patient prognosis [9,10,11].

Pattern recognition performed by a radiologist is a subjective evaluation, but several studies have shown that the accuracy of radiomic features in the discrimination of benign and malignant breast lesions and evaluation prognosis, or response to therapies, can represent an added value to the diagnostic task [12,13,14,15,16,17,18,19]. Automatic recognition tools can provide objective information to support clinical decisions by acting as a second reader in screening while improving the radiologist’s confidence in the challenging diagnostic task [20,21]. Consequently, the radiomics can be useful for predicting important parameters of cancer development such as tumor histological subtype [22] and grading [23], and also for predicting response to cancer treatments [24] through the evaluation of Background Parenchymal Enhancement (BPE) or peri-tumoral tissue of the tumor type [8,10,25]. 

Several approaches to radiomic analysis for different breast imaging modalities are thoroughly described in the literature. In a recent review paper [26], the authors outline the steps in radiomics used for oncology, specifically addressing applications for breast cancer patients and focusing on technical issues. Most papers deal with magnetic resonance (MRI) and the correlation between radiomic tumor features and the clinical data or histopathologic parameters. In another study [27], the authors presented a radiomic analysis of Volumes Of Interest (VOI) in MR images to predict molecular subtype, metastasis of lymph nodes, lymph vessel involvement, grading, hormonal receptor status, Ki67 and HER2 expression of breast lesions. The definition of new predictive models for the prediction of the histological and molecular characteristics of tumors based on radiomic analysis is a very interesting ongoing field of study that is providing encouraging results: this can play a key role in the prognostic evaluations that are crucial for oncological therapeutic decision-making.

This type of study carried out on CESM is very rare, despite this technique having unique characteristics that allow it to simultaneously analyze data derived from mammographic images and those related to tumor neo-angiogenesis.

This makes the analysis of CESM images through its features especially interesting, and could objectify the radiologist’s indications by adding quantitative data to qualitative specialist observations.

Our work aims to highlight the relationship between the radiomic features extracted by CESM images and the histological outcome of breast cancer. Moreover, the discrimination power of the same statistical feature was investigated to predict the differentiation of molecular receptor status, Ki67 levels, tumour grade and two particular cancer subtype molecules, namely HER2-positive and triple-negative.

## 2. Materials and Methods

### 2.1. Experimental Data

From March 2017–June 2018, CESM images of patients referred to Istituto Tumori “Giovanni Paolo II” of Bari (Italy) from the breast cancer screening phase were collected. Patients undergoing CESM had indications for breast MR, but this could not be performed due to several contraindications, or inability to access the exam. CESM investigations were performed on symptomatic or asymptomatic patients with confirmed histological diagnosis of breast cancer obtained by biopsy.

Following the European Society of Breast Imaging (EUSOBI) guidelines [28], CESM for patients with indication to perform breast MRI for staging, but when this could not be carried out due to contraindications or access difficulties, was performed. Our observational study was approved by the Scientific Board of the Institute, and written informed consent prior to CESM examination was signed by all eligible patients. 

A total of 52 patients aged between 37 and 80 years (with a median and first and third quartile of 50, 45.75 and 60.25 years, respectively) were included in the study, resulting in patients being positive for the presence of at least one significant finding after histological survey. 68 primary and, if present, secondary lesions were manually segmented by two breast radiologists. The study was approved by the Institute’s Medical Ethics Committee. Moreover, all eligible patients signed informed consent prior to taking the CESM exam.

### 2.2. CESM Examination 

CESM examination consists of the acquisition of digital mammograms by the dual-energy technique after the administration of an intravenous iodinated contrast medium (CM). Moreover, a combined mammographic image, where only the CM is highlighted, is produced. 

**Figure 1:**
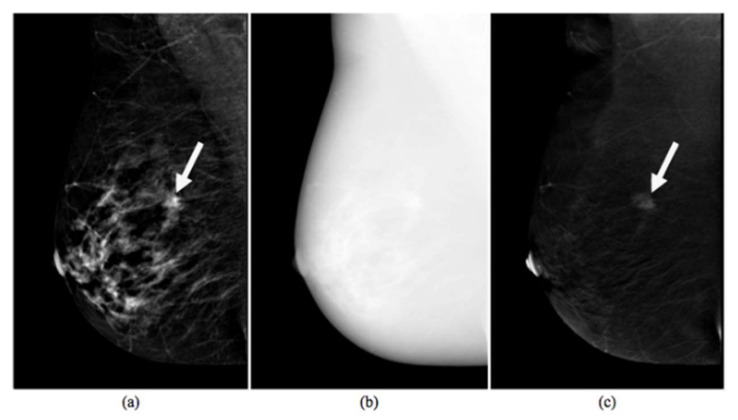
Images produced by Contrast-Enhanced Spectral Mammography (CESM) instrument. Typical example of low-energy (**a**), high-energy (**b**) and recombined (**c**) images [5]. The white arrow points to a suspicious lesion.

A typical example is shown in Figure 1, where a first low-energy image (LE) is completely superimposable on a 2D digital mammographic image (a); a second high-energy image (HE) not visible in the reporting monitor is evident (b); and a third ReCombined (RC) image, obtained through a subtraction algorithm between the first two images, provides information on the tumor microcirculation.

For our study, a modified digital mammography system derived from a standard Senographe Essential (GE Healthcare) was used. Firstly, with an automated injector, a single intravenous injection of 1.5 mL/kg of body mass iodinated contrast agent (Visipaque 320 mg I/mL) was performed at a flow rate of 2–3 mL/s. After two minutes, a set of images was acquired in quick succession. While the breast remained compressed, first the contralateral breast was acquired, then the breast with the suspected lesion. With both axial (CC) and oblique (MLO) views, the CESM examination obtained two images: a low-energy (LE) acquisition at 26–30 kVp and a high-energy (HE) acquisition at 45–49 kVp, depending on breast density and thickness. An anti-allergic premedication was provided for subjects with confirmed allergic diathesis, and despite this two cases of allergic skin reaction after administration of iodinated contrast medium were observed.

CESM images were all in DICOM (Digital Imaging and COmmunications in Medicine) format and were evaluated by two dedicated radiologists with more than ten years of experience in reading mammography and breast MR images. They identified and manually outlined the ROIs containing primary and, if present, secondary lesions. All ROIs were extracted from both LE and RC images.

### 2.3. Histological Outcome

The histological examination was performed through multiple biopsy sampling with 14–16 G core biopsy under ultrasound guidance. The picked whips were inserted in a container with formalin and sent on the same day to the Pathological Anatomy Department. The histological grade and the expression of estrogen receptor (ER), progesterone receptor (PR), Human Epidermal growth factor Receptor 2 (HER2) and Ki-67 antigen associated with cell proliferation were determined by immunohistochemical analysis carried out by the subspecialty department of breast disease in our institute. Specifically, for each sample, the expression of ER, PR and Ki67 were valued in percentage terms. The tumor grade G was defined by the Elston-Ellis modification of the Scarff-Bloom-Richardson grading system on a three-grade scale. Specifically, based on duct structures, the size and shape of the nucleus in the tumor cells and mitotic rate were assessed, and a lesion can be of grade G1 (low grade), G2 (intermediate grade) or G3 (high grade), where a lower grade indicates a better prognosis [29].

### 2.4. Radiomic Analysis

Radiomic analysis was performed on regions of interest (ROIs) manually selected by two radiologists who were experts in quantitative image analysis. A total of seven statistical features were extracted from each original ROI from both LE and RC images, that is, mean, variation coefficient (VC, defined as the ratio of the standard deviation to the mean), difference of maximum and minimum of gray-level values, skewness, entropy, relative smoothness and kurtosis. Therefore, 14 features were obtained for each ROI.

These descriptors are common statistical features that were highly informative in the characterization of tumor tissues in CESM images [12,13]. Mean and variation coefficients and difference of maximum and minimum describe the average intensity and the variability of the gray-level distribution. Entropy is a first-order statistical feature describing the gray-level distribution of the image. The sharpness of the histogram is described by the kurtosis, relative smoothness and skewness. Details of the mathematical notations of these texture features have been previously published [14]. 

### 2.5. Statistical Analysis 

Firstly, in order to evaluate the association between each radiomics feature and clinical and prognostic parameters, a Pearson correlation test for ER (%), PR (%) and Ki67 (%) measured on an interval scale, and a non-parametric Mann–Whitney test for the histological grade (G) and HER2, measured on an ordinal and nominal scale, respectively, were performed. Bonferroni correction was used to adjust for multiple comparison.

Then, six classification tasks were estimated: ER+/ER− (using a cut-off of 1%), PR+/PR− (using a cut-off of 20%), HER2+/HER2−, TN/NTN (non-triple-negative, that is, presence of at least one hormonal receptor expression), Ki67+/Ki67− (using a cut-off of 20%) and High-Grade/Low-Grade (high G3 and low G1-G2). 

A multivariate Linear Discriminant Analysis (LDA) was performed to explore the discriminating power of the overall feature sets. The performance of the prediction model was evaluated using Leave One Out (LOO) cross-validation [30].

All the analysis algorithms used in this work were implemented in MATLAB2018a (MathWorks, Inc., Natick, MA, USA) software.

## 3. Results

Our results show a correlation between CESM image radiomic data and molecular parameters in 68 malignant breast lesions.

Table 1 describes histological characteristics, grading, tumor size and lymph node involvement. 

Table 2 shows the correlation coefficients between the radiomic features and molecular receptor status and Ki67 levels. No radiomic features extracted from low-energy images are significantly correlated with the proliferative activity of Ki67, while the hormonal receptor ER is significantly correlated with variation coefficient (LE_VC) and variation range (LE_Max-Min) and PR with skewness index (LE_Skewness). Regarding radiomic features calculated on the recombined image, no radiomic features are significantly correlated with the hormonal receptor PR, whereas ER is significantly correlated with variation coefficient (RC_VC) and variation range (RC_Max-Min). Ki67 level is significantly correlated with variation coefficient (RC_VC), variation range (RC_Max-Min), entropy (RC_Entropy) and relative smoothness (RC_RelativeSmoothness) indices. 

Moreover, it was found that HER2 is significantly associated with relative smoothness calculated on ROIs of LE images (*p*-value Mann-Whitney test < 0.05), whereas grading G is statistically associated with variation coefficient, entropy and relative smoothness calculated on ROIs of RC images (*p*-value Mann-Whitney test < 0.05).

**Figure 2:**
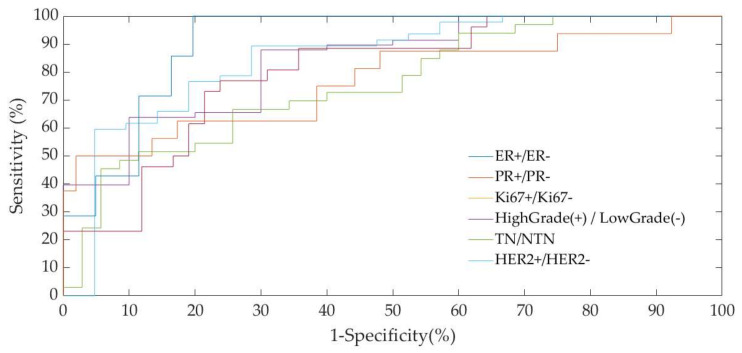
Area Under the Curve (AUC) of the Receiver Operating Characteristic (ROC) of the multivariable discriminant analysis model for each classification task.

Figure 2 shows the Area Under the Curve (AUC) of the Receiver Operating Characteristic (ROC) of the multivariable discriminant analysis model for each classification task evaluated with Leave One Out (LOO) cross-validation. Specifically, as summarized in Table 3, for the tasks ER+/ER−, PR+/PR−, HER2+/HER2−, Ki67+/Ki67−, High-Grade/Low-Grade and TN/NTN, the discrimination model achieved AUC values of 82.76%, 82.13% 83.48%, 79.85%, 76.80% and 90.89%, respectively (Table 3).

## 4. Discussion

The radiomic analysis of tumor features extracted from CESM images combined with qualitative and quantitative information on morphology and functionality represents an important tool for breast tumor characterization.

In our work, the discrimination power of the same statistical feature was investigated to predict histological outcomes and two particular subtypes of tumors, namely HER2-positive and triple-negative. Our work showed encouraging results for the differentiation between ER+/ER−, PR+/PR−, HER2+/HER2−, Ki67+/Ki67−, High-Grade/Low Grade and TN/NTN. In particular, the highest performances were obtained for discriminating HER2+/HER2− (90.87%), ER+/ER− (83.79%) and Ki67+/Ki67− (84.80%).

CESM is studied less than MRI as it is more recent and less common, but it lends itself very well to radiomic analysis, presenting a set of characteristics that make it unique among methods in breast cancer: the possibility of simultaneously studying mammographic features and features deriving from tissue neo-angiogenesis on the same patient provides the examiner with a large amount of diverse information, much more than with traditional techniques.

Studies of CESM have essentially focused on imaging analysis with an auxiliary system for reporting, and on radiomic features for benignant–malignant differentiation [12,13]. 

Recent works published in the literature have aimed at investigating the potential of CESM radiomic analysis in the differentiation of tumors, but there are very few that evaluate correlation with different molecular subtypes.

In one study in particular [23], accuracy was high in most comparisons (87.4% for the differentiation between invasive and non-invasive carcinoma, 100% triple-negative vs. triple-positive, 82.1% triple-negative vs. HR+, 97.2% HER+/HR+ vs. HER2-/HR+, 90% G1 vs. G2/G3 for invasive carcinoma, 100% G1 vs. G2/G3 for non-invasive carcinoma).

In another study [31], the potential of CESM radiomic analysis for the non-invasive differentiation of invasive breast cancer, hormone receptor status and tumor grade was investigated.

An interesting study [6] investigated and compared the potential of radiomics analysis of CESM and MRI of the breast for the non-invasive assessment of tumor invasiveness, hormone receptor status and tumor grade in patients with primary breast cancer.

In order to improve specificity, Liu et al. [7] quantitatively assessed enhancement intensity of breast lesions of different pathological types and hormonal receptor status and evaluated the consistency of enhancement patterns comparing CESM and MRI.

CESM is a recent diagnostic investigation technique, and there are therefore few radiomics studies in this regard. However, several studies in the literature have investigated the usefulness and reliability of radiomics analysis of different images, demonstrating that it could potentially improve diagnosis and characterization of lesions. Li et al. [32] showed an improvement in performance for the characterization of benign or malignant lesions through the radiomic analysis of FFDM. This was done by comparing tumoral features with texture analysis of the parenchyma of the healthy contra-lateral breast.

Tagliafico et al. have shown a correlation between tissue features and some histological characteristics of breast cancer with the use of tomosynthesis [33,34]. In the first study [33], they differentiated between women with proliferation index (Ki67) higher or lower than 14% through imaging function. In the second study, they identified some parameters strongly correlated to cancer presence, tumor dimensions and estrogenic receptor status. This study was conducted on dense patterns [34] by extracting features on normal tissue from neoplastic cohorts and control healthy cohorts. 

Nevertheless, a large number of the studies of radiomic analysis of breast cancer was conducted on MRI. This technique is multi-parametric and allows for several observations, both morphological and based on tumor contrast kinetics. Several studies using this technique showed a correlation between molecular subtypes and qualitative and semi-quantitative tumor features [24,35,36] and between perfusion parameters [37] and semi-automatic features of the images [9,22,38,39,40,41,42,43] after fusion imaging [44].

Some works [23,36] have studied the correlation between radio-genomic features and clinical variables (pathologic stage, receptors, lymph nodes metastasis, grading), while others studied the correlation between features of the tumor, of the healthy surrounding parenchyma and of the healthy contra-lateral breast on MR images, along with histologic features. The evaluation of the tumoral region as a whole and related features reflects the tumor complexity and heterogeneity: correlation with histologic features improves when the peritumoral tissue is also considered. This has been confirmed by studies of the importance of the local tumoral microenvironment for the evaluation of the progression and of the biologic aggressiveness over time in breast cancer [10,45].

Another important observation is that most automated systems for radiomic analysis are not affected by factors such as breast density or background parenchymal enhancement (BPE), which may be limiting for human reporting capabilities [17,46]. Consequently, these systems also preserve their reliability in more complex breast investigations.

The CESM technique has the same potentialities as MRI when iodized contrast medium is used. In this case, the levels of diagnostic accuracy, sensibility, specificity and positive or negative predictive values are similar [4]. Nevertheless, the CESM technique was better tolerated by patients [47].

Presently, the determination of the biological and molecular features for malignancies is performed with biopsy. Unfortunately, this examination is invasive and is not always able to detect tumor heterogeneity at the diagnosis nor changes over time. This is even more so for large malignancies [35,48]. For these reasons, there is great interest in alternative, non-invasive and cheaper methods to derive the same information directly from the radiologic images.

In this context, the radiomic analysis of tumor features extracted from CESM images combined with qualitative and quantitative information on morphology and functionality represent an important tool for breast tumor characterization.

According to the present study, the CESM technique is particularly suitable for this kind of analysis. In fact, it is possible to examine tumor features simultaneously on morphologic mammographic-like images (LE) and on ReCombined images (RC), from which information can be extracted about the contrast medium diffusion in the tumorous microcirculation. If our preliminary results are confirmed by studies on larger cohorts, they would be extremely important as they would allow the recognition of aggressive tumor types, which are frequently treated with neoadjuvant therapies.

## 5. Conclusions

Our preliminary results confirm that histological outcomes and some molecular subtypes of tumors (mainly HER2+ tumors) can be differentiated with satisfactory accuracy by means of statistical features extracted from CESM images. Therefore, the radiomic analysis of this novel diagnostic instrument can play a key role in early detection, in control of the disease over time and in response to treatments for the reported subtypes of tumors, especially for multiple forms.

A limitation of the study is the reduced size of the cohort. Even though the adopted cohort is consistent with almost all the radiomic studies in the literature, future works should include both validation studies, in order to test the robustness of the results in a larger population, and an analysis of other types of features.

Moreover, the evaluation of the changes in tumor features as a consequence of oncological treatment can be a future development building on the results of this study. This could be achieved through the recognition of biomarkers for the prediction of response to treatment and the differentiation of responder and non-responder patients.

## Figures and Tables

**Table 1:** Characteristics of the 68 tumors analyzed in the study.

Characteristic	No. of Lesions (%)
Histological subtype	
Invasive ductal carcinoma	57 (83.82%)
Infiltrating lobular carcinoma	9 (13.24%)
Ductal carcinoma in situ	2 (2.94%)
Histological grade	
High (G1)	14 (20.59%)
Intermediate (G2)	28 (41.18%)
Low (G3)	26 (38.23%)
Tumor size	
<10 mm	34 (50.00%)
10–19 mm	25 (36.76%)
20–29 mm	1 (1.47%)
≥30 mm	1 (1.47%)
Unknown	7 (10.30%)
Lymph node state	
Node-negative	29 (42.65%)
Node-positive	29 (42.65%)
Unknown	10 (14.70%)

**Table 2:** Correlation between radiomic features and estrogen receptor (ER), progesterone receptor (PR) and Ki67.

**Features**	**LE_Mean**	**LE_VC**	**LE_Max-Min**	**LE_Skewness**	**LE_Entropy**	**LE_Relative Smoothness**	**LE_Kurtosis**
ER (%)	0.10	−0.23 *	−0.23 *	−0.07	0.01	−0.16	0.02
PR (%)	−0.07	−0.07	−0.14	−0.35 ***	−0.04	−0.10	−0.14
Ki67 (%)	−0.02	0.14	0.20	−0.01	0.02	0.11	−0.13
**Features**	**RC_Mean**	**R_VC**	**RC_Max-Min**	**RC_Skewness**	**RC_Entropy**	**RC_Relative Smoothness**	**RC_Kurtosis**
ER (%)	0.00	−0.26 **	−0.21 *	−0.11	−0.13	−0.20 *	0.17
PR (%)	−0.07	−0.15	−0.10	−0.03	−0.15	−0.18	0.05
Ki67 (%)	0.15	0.28 **	0.31 **	0.08	0.28 **	0.29 **	−0.17

* *p*-value < 0.10, ** *p*-value < 0. 05, *** *p*-value < 0.01.

**Table 3:** Class size and AUC values resulting from six classification tasks.

	(Pos/Neg)	AUC
ER+/ER−	58/10	83.79%
PR+/PR−	33/35	75.50%
Ki67+/Ki67−	47/21	84.80%
High-Grade(+)/Low-Grade(−)	26/42	79.85%
TN/NTN	7/61	76.80%
HER2+/HER2−	16/52	90.87%
